# Welche Rolle spielen Kinder in Schulen und Kindertagesstätten bei der Übertragung von SARS-CoV-2? – Eine evidenzbasierte Perspektive

**DOI:** 10.1007/s00103-021-03454-2

**Published:** 2021-11-18

**Authors:** Anna Kern, Jessica Diebenbusch, Reinhard Berner, Ingeborg Krägeloh-Mann, Freia De Bock, Herbert Renz-Polster, Johannes Hübner

**Affiliations:** 1 Kinderklinik und Kinderpoliklinik im Dr. von Haunerschen Kinderspital, LMU Klinikum, München, Deutschland; 2 Klinik und Poliklinik für Kinder- und Jugendmedizin, Universitätsklinikum und Medizinische Fakultät Carl Gustav Carus, TU Dresden, Dresden, Deutschland; 3 IKM Abt. Neuropädiatrie, Entwicklungsneurologie, Sozialpädiatrie, Universitätsklinik für Kinder- und Jugendmedizin Tübingen, Tübingen, Deutschland; 4 Bundeszentrale für gesundheitliche Aufklärung (BZgA), Köln, Deutschland; 5 Mannheimer Institut für Public Health, Universität Heidelberg, Mannheim, Deutschland

**Keywords:** COVID-19, Pandemie, Kinder, Vektor, Schule, COVID-19, Pandemic, Children, Disease vector, Schools

## Abstract

Sind Kinder und Jugendliche relevante Vektoren für die Übertragung von SARS-CoV-2? Und welche Rolle spielt es, wenn sie eine Schule oder Kindertagesstätte besuchen? Diese Fragen konnten zu Beginn der Pandemie nur unzureichend beantwortet werden. So wurden weltweit Schulen und Kinderbetreuungseinrichtungen geschlossen, um die Verbreitung von SARS-CoV‑2 einzudämmen. Inzwischen ist die Rolle von Kindern im Gesamtgeschehen der Pandemie jedoch klarer. Die Rate von SARS-CoV-2-Infektionen bei Kindern unter 10 Jahren war im Jahr 2020 deutlich niedriger als die bei Erwachsenen. Zudem zeigte sich bei Kindern ein deutlich milderer Verlauf der Erkrankung.

Analysen zu Ausbrüchen an Schulen und Kinderbetreuungseinrichtungen kamen mehrheitlich zu dem Ergebnis, dass die Weitergabe des Virus in den Einrichtungen zwar stattfindet, jedoch das Infektionsgeschehen insgesamt nicht maßgeblich beeinflusst. Trotz dieser Erkenntnisse hält die deutsche Politik Schulschließungen weiterhin für einen integralen Baustein der Pandemiebekämpfung, wohingegen viele Fachgesellschaften, wie die Deutsche Gesellschaft für Pädiatrische Infektiologie e. V. (DGPI), betonen, dass es sich um das letzte Mittel in der Bekämpfung der Pandemie handeln sollte. Diese Botschaft hat auch eine evidenzbasierte und auf interdisziplinärem Expertenkonsens aufgebaute S3-Leitlinie, die bereits Anfang Februar 2021 klare Empfehlungen für Zeiten hoher Inzidenzen in der Gesamtbevölkerung ausgesprochen hat, die Schulschließungen nur noch in Ausnahmefällen für notwendig erachten.

In diesem Artikel möchten wir die Datenlage mit Stand Juni 2021 zu diesem Thema darlegen, einen Blick in die Zukunft wagen und diskutieren, unter welchen Umständen ein regulärer Präsenzunterricht gelingen kann, ohne das Risiko einer unkontrollierten Ausbreitung von SARS-CoV‑2 in Kauf nehmen zu müssen.

## Abstract (en)

Are children and adolescents relevant disease vectors when it comes to the transmission of SARS-CoV-2? Moreover, do they play a role as relevant disease vectors in a school or kindergarten setting? These questions could not be sufficiently answered at the beginning of the pandemic. Consequently, schools and childcare facilities were closed to stop the spread of SARS-CoV‑2. Over the past few months, researchers have gained a more detailed understanding of the overall pandemic situation. The SARS-CoV‑2 infection rate in children below 10 years of age in 2020 has been substantially lower than in adults. In addition, it showed that children had a milder course of disease.

Although a majority of the analyses performed in schools and childcare facilities revealed that the virus is transmitted in these facilities, these transmissions did not, however, have a considerable influence on the overall rate of new infections. Despite these findings, German politicians continue to advocate for the closure of childcare facilities, including schools, to fight the pandemic, whereas many specialist societies such as the German Society for Pediatric Infectious Diseases (DGPI) have emphasized that such closures should be the measure of last resort in combating the pandemic. The same message is also conveyed by a German evidence-based S3 guideline established by an interdisciplinary expert group that had already put forward clear recommendations for high incidences in the general population at the beginning of February 2021, indicating that school closures were only required in exceptional cases.

In this article, we would like to outline the situation based on the currently available data, try to predict the future, and discuss the circumstances necessary to realize normal classroom teaching without accepting the risk of an uncontrolled spread of SARS-CoV‑2.

## Einleitung

Sind Kinder und Jugendliche relevante Vektoren für die Übertragung von SARS-CoV‑2 („severe acute respiratory syndrome coronavirus type 2“)? Und wie wirkt sich dies aus, wenn Kinder eine Schule oder Kindertagesstätte besuchen? Das sind Fragen, die sowohl die Wissenschaft als auch die Medien und Politik seit Beginn der Pandemie im März 2020 beschäftigen.

Da hier anfangs und insbesondere auch zu Beginn der dritten Pandemiewelle im Winter und Frühjahr 2021 angesichts von Virusmutationen große Unklarheit herrschte, wurden weltweit Schulen geschlossen, um so das Infektionsgeschehen einzudämmen. Inzwischen sind die Übertragungswege von SARS-CoV‑2 und damit auch die infektionsepidemiologische Rolle von Kindern klarer geworden [1–6]. Kinder unter 10 Jahren schienen im Jahr 2020 insgesamt seltener mit SARS-CoV‑2 infiziert zu sein als Erwachsene [1]. Auf ein geringeres Risiko von Kindern, sich anzustecken, deuten Studien zur Übertragung in privaten Haushalten zu Beginn der Pandemie hin [2, 3].

Eine Analyse zu Ausbrüchen an Schulen und Kinderbetreuungseinrichtungen in Deutschland kam zu dem Ergebnis, dass die Weitergabe des Virus in den Einrichtungen zwar stattfindet, jedoch das Infektionsgeschehen insgesamt hierdurch nicht maßgeblich beeinflusst wird [7]. Diese Einschätzung wird auch in einer Stellungnahme der Deutschen Gesellschaft für Pädiatrische Infektiologie e. V. (DGPI) zur Rolle von Schulen und Kindertagesstätten in der COVID-19-Pandemie vertreten [8].

Trotz all dieser Erkenntnisse hielt (und hält) die deutsche Politik bei hohen Zahlen von positiven SARS-CoV-2-PCR-Tests in der Bevölkerung (im Weiteren als „Inzidenz“ bezeichnet) am Konzept des Distanzunterrichts fest und öffnet Bildungseinrichtungen bisher nur zögerlich für den Regelbetrieb. Dies scheint insbesondere auch bedauerlich angesichts der Tatsache, dass eine evidenzbasierte und auf interdisziplinärem Expertenkonsens aufgebaute S3-Leitlinie bereits Anfang Februar 2021 klare Empfehlungen für Zeiten hoher Inzidenzen in der Gesamtbevölkerung ausgesprochen hat, die Schulschließungen nur noch in Ausnahmefällen notwendig machen [9].

In diesem Artikel möchten wir deshalb diskutieren, welche Rolle Kinder und Jugendliche bei der Übertragung von SARS-CoV‑2 spielen, wenn sie eine Schule oder Kindertagesstätte besuchen. Hierzu soll die aktuelle Datenlage mit Stand Juni 2021 beleuchtet werden. Zudem möchten wir auf mögliche Schwierigkeiten bei der Interpretation dieser Daten hinweisen und aufzeigen, wie zukünftige Studien gegebenenfalls angelegt sein sollten, um noch aussagekräftigere Ergebnisse zu liefern. Nicht zuletzt möchten wir darauf eingehen, unter welchen Umständen ein regulärer Präsenzunterricht gelingen kann, ohne das Risiko einer unkontrollierten Ausbreitung von SARS-CoV‑2 in Kauf nehmen zu müssen.

Einschränkend zur Bewertung der Rolle von Kindern und Jugendlichen in der Pandemie muss an dieser Stelle erwähnt werden, dass sich die infektionsepidemiologische Lage aufgrund des Auftretens neuer Varianten stetig ändert. Bei Erstellung des Artikels war in Deutschland die Alphavariante vorherrschend. Zu der dann aufkommenden Deltavariante kann der Artikel aufgrund der bis dato unzureichenden Datenlage nur allgemeine Aussagen treffen. Es ist jedoch zu erwarten, dass sich die Möglichkeiten der Prävention auch mit dem Auftreten neuer Varianten nicht grundlegend ändern werden; sowohl die Weltgesundheitsorganisation (WHO) als auch das Europäische Zentrum für die Prävention und die Kontrolle von Krankheiten (ECDC) haben darauf hingewiesen, dass neue Varianten (die auch weiterhin zu erwarten sind) die Notwendigkeit von Schulunterricht in Präsenz nicht infrage stellen dürfen.

## Inzidenz und Erkrankungsverlauf von SARS-CoV-2-Infektionen bei Kindern

Wie im Jahr 2020 erfasste Daten aus Europa zeigen, scheinen vor allem jüngere Kinder unter 10 Jahren im Vergleich zur Allgemeinbevölkerung weniger von einer SARS-CoV-2-Infektion betroffen zu sein [1, 10].

Kinder scheinen sich nicht nur seltener zu infizieren, sie erkranken auch meist nur mild an COVID-19. Zu diesem Ergebnis kam unter anderem eine Erhebung des ECDC im Zeitraum vom 01.08. bis 29.11.2020, bei der 1,8 Mio. gemeldete Fälle einer SARS-CoV-2-Infektion auf die Schwere des Verlaufs nach Altersgruppen getrennt untersucht wurden [10]. Auch Daten des Robert Koch-Instituts (RKI) aus Deutschland belegen dies [11]. Schwere oder letale Verläufe sind in der Altersgruppe der Kinder und Jugendlichen bis 18 Jahre insgesamt eine Rarität ebenso wie eine Hospitalisierung.

Welche Gründe es für die bisher erfasste niedrigere Inzidenz und den milden Verlauf bei jüngeren Kindern geben könnte, soll in den folgenden Abschnitten erläutert werden.

## Vermehrt milde oder asymptomatische Verläufe bei Kindern

Ein Grund für die Annahme, dass Kinder nicht so häufig mit einer SARS-CoV-2-Infektion diagnostiziert werden, könnte auf eine hohe Anzahl sehr milder bzw. asymptomatischer Verläufe zurückzuführen sein.

Eine Metaanalyse bisheriger Studien schätzt den Anteil gänzlich asymptomatischer SARS-CoV-2-Infektionen bei Erwachsenen auf rund 20 % [12]. Die Studienlage hierzu ist jedoch heterogen. Einige Studien gehen davon aus, dass besonders bei Kindern und Jugendlichen die Anzahl der gänzlich asymptomatischen Fälle höher liegt als bei Erwachsenen [10]. Schätzungen, die eine hohe Anzahl asymptomatischer Fälle innerhalb der Altersgruppe der Kinder und Jugendlichen vermuten, wurden in SARS-CoV-2-Antikörperstudien ermittelt. Bei einer solchen in Deutschland durchgeführten Studie mit ca. 15.000 Kindern bis 18 Jahre aus dem Jahr 2020 lag die Anzahl der seropositiven Kinder um das 6‑Fache höher im Vergleich zu den in diesem Zeitraum offiziell gemeldeten Zahlen [13]. Zu Beginn des Jahres 2021 lag die Anzahl der seropositiven Kinder noch um das 3‑ bis 4‑Fache höher im Vergleich zu den in diesem Zeitraum gemeldeten Zahlen [14]. Nahezu 50 % der seropositiven Kinder gaben an, zum betreffenden Zeitpunkt asymptomatisch gewesen zu sein. Zu berücksichtigen ist allerdings, dass in diesem Zeitraum gerade bei Kindern sehr häufig keine Erregerdiagnostik durchgeführt wurde.

## Geringeres Ansteckungsrisiko

Eine weitere Erklärung für die niedrigere Inzidenz bei Kindern unter 10 Jahren könnte ein niedrigeres Ansteckungsrisiko sein. Schon zu Beginn der Pandemie hat eine Studie, die die Übertragung der Infektionen in privaten Haushalten in China untersuchte, gezeigt, dass die Infektionsrate bei Kindern 4 % betrug, bei Erwachsenen jedoch 17,1 % [2]. Zudem konnten Kinder nur selten als Indexfälle^1^ identifiziert werden [2]. Zu einem ähnlichen Ergebnis kommt eine Metaanalyse. Hier lag die sekundäre Infektionsrate in privaten Haushalten insgesamt bei 18,1 % [3]. Erwachsene hatten ein höheres Risiko sich zu infizieren als Kinder und Jugendliche unter 18 Jahren (relatives Risiko, RR 1,71), besonders Ehepartner zeigten höhere Infektionsraten im Vergleich zu anderen Kontakten des Haushalts (RR 2,39). Es muss jedoch darauf hingewiesen werden, dass die Studienlage sich zum aktuellen Zeitpunkt auf Studien und Metaanalysen bezieht, die zu Beginn der Pandemie durchgeführt wurden. So könnten die Ergebnisse auch den Effekt von Schulschließungen und geltenden Kontaktbeschränkungen widerspiegeln.

Dass auch immunologische Faktoren eine geringere Ansteckungswahrscheinlichkeit und auch Infektiosität von Kindern begründen könnten, lassen Studien an Zellkulturen vermuten, nach denen die Vermehrungsfähigkeit der von Kindern im Nasopharynx (Nasenrachenraum) abgestrichenen Viren in lebenden Zellen geringer ist als bei Erwachsenen [15]. Tatsächlich haben besonders Kinder im Kindergartenalter häufig virale Infekte und verfügen so über ein gut „trainiertes“ Immunsystem; die Bildung kreuzreaktiver Antikörper auf Coronaviren könnte dabei eine Rolle spielen, da dadurch eine SARS-CoV-2-Infektion schon bei Eintritt der Viren in den Körper bekämpft werden könnte [16].

## Geringeres Übertragungsrisiko durch Kinder

Der häufig milde bzw. asymptomatische Krankheitsverlauf bei Kindern und Jugendlichen könnte auch bedingen, dass sie weniger ansteckend sind. Verschiedene Studien weisen darauf hin, dass symptomatische im Vergleich zu asymptomatischen Personen ein höheres Risiko haben, andere anzustecken [3, 12]. Es zeigte sich, dass Menschen mit milden Verläufen sowohl eine geringere als auch eine kürzere Periode der Infektiosität aufwiesen [17]. Auch waren sekundäre Infektionsraten, die von milden Verläufen ausgingen, geringer [3]. Zudem konnte in einer Studie nachgewiesen werden, dass die Viruslast bei Folgefällen im Vergleich zum Indexfall niedriger war [18]. Wie ansteckend manifest an COVID-19 erkrankte Kinder im Vergleich zu Erwachsenen sind, ist eine bis heute offene Frage.

Die infektionsepidemiologische Rolle von Kindern wurde und wird häufig mit Ergebnissen aus Laboruntersuchungen begründet, in denen die Virenlast bei infizierten Individuen per Abstrich aus dem Nasenrachenraum (Naso- bzw. Oropharynx) gemessen wird. Tatsächlich liegen Ergebnisse vor, nach denen die so gemessene Virenlast bei infizierten Kindern ähnlich hoch sein könnte wie die bei infizierten Erwachsenen [19, 20]. Die vergleichende Interpretation von in Abstrichen gemessener Virenlast ist allerdings schwierig, da die gemessenen Werte stark vom Zeitpunkt der Probenentnahme im Infektionsverlauf abhängen, der sich zwischen verschiedenen Altersgruppen oft systematisch unterscheidet. Eine Studie, die diesen Faktor berücksichtigen konnte, weist Kindern eine deutlich geringere Virenlast zu [21].

Die Menge des Virusmaterials, welches im Zuge einer Infektion mit SARS-CoV‑2 weitergegeben wird, hängt jedoch nur in Teilen von der gemessenen Virenlast im Naso- bzw. Oropharynx ab. Sie ist auch davon abhängig, wie gut eine infizierte Person das Virus verbreiten kann. Inzwischen ist eindeutig belegt, dass sich das Virusmaterial im Rahmen der Übertragung mittels Tröpfchen bzw. Aerosolen im Raum verbreitet, wobei der Fähigkeit, Aerosole zu produzieren, die entscheidende Rolle zugesprochen wird [22]. In experimentellen Studien konnte gezeigt werden, dass die Ausscheidung von Aerosolen vom Body-Mass-Index, dem Stadium der Infektion und – vor allem – dem Alter abhängig ist, wobei Kinder deutlich weniger Aerosole produzieren als Erwachsene [23–25]. Dies dürfte auch der kindlichen Anatomie zuzuschreiben sein (geringerer Durchmesser und Länge der Atemwege, verminderte Kraft des Hustenstoßes; [24]). Zudem werden bei pädiatrischen COVID-19-Infektionen weniger Fälle mit Husten oder der Entwicklung einer COVID-19-assoziierten Pneumonie berichtet [26], wodurch aufgrund des fehlenden Hustens auch weniger Aerosole produziert werden dürften.

## Kaum Beteiligung von Kindern an Superspreading-Events

Die SARS-CoV-2-Pandemie wird – anders als Influenzapandemien – von nur wenigen stark übertragenden Personen unterhalten. Es wird angenommen, dass nur 2 % der infizierten Individuen 90 % der Viren verteilen [4]. Vielüberträger („Superspreader“) unterscheiden sich von Wenigüberträgern vor allem im Alter, im sozialen Verhalten (z. B. hohe Mobilität in breiten, offenen Kontaktnetzen) sowie in biologischen Faktoren (z. B. Virenlast und Ausscheidungsfähigkeit von infektiösem Material; [5, 6]). Sogenannte Superspreading-Events (Superverbreitungsereignisse) sind zwar auch in pädagogischen Einrichtungen möglich, bisher scheinen Kinder allerdings nur selten daran beteiligt zu sein, was auch daran liegen könnte, dass gerade jüngere Kinder sich vor allem in klar definierten, deutlich begrenzten Kontaktnetzen bewegen und vergleichsweise wenig Aerosole produzieren [5].

Entgegen dem Ausbreitungsprofil bei anderen Atemwegserregern scheint also im aktuellen pandemischen Geschehen keine substanzielle treibende Kraft von Kindern auszugehen, obgleich auch hier Übertragungen stattfinden und Ausbruchsgeschehen wirksam verhindert werden müssen [7]. Insbesondere Kinder unter 10 Jahren könnten durch ein geringes Risiko, sich anzustecken und das Virus zu übertragen, sogar eher ein Abbremsen als eine Beschleunigung der Pandemie bewirken [10].

## Die Rolle von Schulen und Kinderbetreuungseinrichtungen in der Pandemie

Die Rolle der Kinder und Jugendlichen in der Pandemie hängt nicht nur von dem Risiko ab, sich selbst zu infizieren oder andere anzustecken, sondern auch von der Anzahl ihrer Kontakte mit Gleichaltrigen oder Erwachsenen. Ein Ort möglicher Kontakte sind Schulen und Kinderbetreuungseinrichtungen. Ob hier relevante Übertragungen von SARS-CoV‑2 stattfinden, wurde inzwischen in mehreren Ländern untersucht [27–30].

Das ECDC hat anhand der Daten aus 12 europäischen Ländern Ausbrüche von COVID-19 innerhalb des Schulbetriebes analysiert [10]. Die meisten Ausbrüche führten zu Clustern von unter 10 Personen und die meisten Übertragungen fanden in weiterführenden Schulen statt. In den Ländern, in denen Kontaktpersonennachverfolgung praktiziert wurde, zeigte sich, dass die Übertragungen in Schulen weniger als 1 % der Fälle aller Übertragungen ausmachten [10].

Auch Studien aus anderen Ländern kommen zu dem Ergebnis, dass Übertragungen innerhalb des Schulbetriebs nicht häufig sind [10]. Eine Fallkontrollstudie aus den USA mit 397 pädiatrischen Patienten mit SARS-CoV-2-Infektion zeigte, dass Präsenzunterricht in den Schulen nicht mit einer erhöhten Wahrscheinlichkeit einer SARS-CoV-2-Infektion einherging [31]. Im Rahmen dieser Studie wurden die Kontakte der Infizierten in den 14 Tagen vor deren Infektion untersucht. Die meisten Infektionen gingen auf private Treffen mit Personen außerhalb des eigenen Haushalts zurück.

In Deutschland sind im Herbst 2020 bei insgesamt sehr hohen Inzidenzwerten Clusterinfektionen in Schulen weitestgehend ausgeblieben [8]. Schulen waren hier größtenteils bis kurz vor den Weihnachtsferien geöffnet.

Das RKI hat bisher (Stand 24.06.2021) lediglich 2,2 % der insgesamt 3,7 Mio. COVID-19-Infektionen in Deutschland in Verbindung mit Schulen und Kindertagesstätten gebracht (Erfassung nach § 33 Infektionsschutzgesetz (IfSG), Stand 24.06.2021). Hierbei entfallen 1,6 % auf Schulen und 0,6 % auf Kindertagesstätten. Bei etwa 10,9 Mio. Schülern und Schülerinnen in Deutschland entspricht das etwa 0,6 % der Schülerschaft [32]. Demgegenüber wurden etwa ebenso viele Infektionen (1,9 %) in Verbindung mit Pflegeeinrichtungen gebracht (Erfassung nach § 36 IfSG; [33]). Da lediglich etwa 731.000 Menschen in Deutschland in einer Pflegeeinrichtung leben, waren hier im Vergleich zu Schülerinnen und Schülern deutlich mehr betroffen, nämlich 9,8 % [34].

Diese Daten werden untermauert durch weitere Studien aus Deutschland. Eine Studie des RKI, die COVID-19-Ausbrüche an Schulen von März bis August 2020 untersuchte, fand nur wenige und mehrheitlich kleine Ausbrüche an Schulen [30]. Eine Studie des Landesuntersuchungsamts Rheinland-Pfalz zusammen mit dem Institut für Global Health der Universität Heidelberg kam zu ähnlichen Ergebnissen. Hier wurde von August bis Dezember 2020 eine systematische Analyse von SARS-CoV-2-Infektionsfällen an Schulen und Kindergärten durchgeführt [27]. Insgesamt wurden 784 unabhängige Indexfälle übermittelt, davon waren bei 441 genaue Daten verfügbar. 360 Indexfälle, also 82 %, verursachten bis Dezember 2020 keinen einzigen Folgefall in den Einrichtungen. Die übrigen 81 Indexfälle führten zu 196 Folgefällen, dies entspricht einer Infektionsrate von 1,34 %. Die sekundäre Infektionsrate in privaten Haushalten liegt hingegen deutlich höher, nämlich bei 18,1 % (wie oben gezeigt). Die niedrige Infektionsrate in Schulen und Kinderbetreuungseinrichtungen bei konsequent umgesetzten (Hygiene‑)Maßnahmen zeigt, dass diese in der Lage sind, Übertragungen zu verhindern. Eher gibt dieser Wert noch eine Überschätzung des Infektionsgeschehens wieder, da nie sicher differenziert werden kann, ob die sekundäre Übertragung in der Schule oder im privaten Kontakt mit Freunden oder Familie stattfand. Zuletzt wurde von einem deutschen Forscherteam aufgrund der Auswertung von bayerischen Schuldaten sogar berechnet, dass die Rückkehr zum Präsenzunterricht mit verpflichtenden Tests einen positiven Einfluss auf den Pandemieverlauf in Deutschland haben könnte [35], da die Teststrategie an Schulen helfen kann symptomlose Infektionen aufzudecken und somit Infektionsketten frühzeitig zu durchbrechen.

## Maßnahmen zur Reduktion der Übertragungswahrscheinlichkeit an Schulen

Insgesamt sind Übertragungen von SARS-CoV‑2 an Schulen zwar nicht häufig, sie sollten aber, wo immer möglich, weiter eingedämmt werden. Im Schulbetrieb hat man deshalb in vielen Ländern strukturiert und flächendeckend Maßnahmen ergriffen, um das Risiko der SARS-CoV-2-Übertragung weitgehend zu minimieren. November und Dezember 2020 haben gezeigt, dass Schulen in Deutschland im „Präsenzunterricht unter Corona-Bedingungen“ betrieben werden können [36]. Hierzu trugen auch die ergriffenen Infektionsschutzmaßnahmen AHA + L (Abstand einhalten, Hygieneregeln beachten, im Alltag eine Maske tragen und Lüften), das Motto „Stay home when sick“ und die differenzierte Kontaktpersonennachverfolgung bei [36]. Seit Ende der Osterferien 2021, in manchen Ländern auch schon früher, wurde zudem ein flächendeckendes Testkonzept mit zweimal wöchentlichen Schnelltests für Kinder im Präsenzunterricht etabliert. Allerdings hatte diese Teststrategie leider nicht zur Folge, dass Schulen auch bei hoher Gesamtinzidenz offengehalten wurden.

Um in Deutschland Hygienekonzepte an Schulen zu optimieren und evidenzbasiertes Handeln zu ermöglichen, wurde im Dezember 2020 sowie Januar 2021 eine S3-Leitlinie „Maßnahmen zur Prävention und Kontrolle der SARS-CoV-2-Übertragung in Schulen“ erarbeitet [9]. Ein Expertenrat verschiedener Fachrichtungen empfiehlt eine Reihe von Maßnahmenpaketen zur Verminderung des Infektionsrisikos. Zu den empfohlenen Maßnahmen im laufenden Schulbetrieb gehört das Maskentragen von Schüler*innen und Schulpersonal. Regelmäßiges und ausreichendes Lüften der Klassenzimmer in bestimmten zeitlichen Abständen ist ein weiterer wichtiger Baustein. Ein einzuhaltender Mindestabstand zwischen Schulkindern sollten nicht nur in der Schule, sondern auch auf Schulwegen im öffentlichen Personennahverkehr und Schulbussen umgesetzt werden.

Ein ebenso hoher Empfehlungsgrad ergibt sich für die Reduzierung der Anzahl von Schülerinnen und Schülern im Präsenzunterricht und/oder der Kohortierung (d. h. geteilte Klassen) abhängig von der regionalen Gesamtinzidenz. Schulschließungen sollten letzte Mittel in der Bekämpfung der Pandemie sein.

## Weitere Faktoren, die das Infektionsgeschehen an Schulen beeinflussen

Hygienekonzepte an Schulen helfen das Infektionsgeschehen vor Ort einzudämmen [49]. Allerdings gibt es weitere Faktoren, die die Infektionsdynamik an Schulen beeinflussen, jedoch nicht direkt mit dem Schulbetrieb zusammenhängen. Diese Faktoren sind im folgenden Abschnitt zusammengefasst, um den Blick für weitere effektive Schutzmaßnahmen zu weiten, die auch die Umgebung der Schulen miteinschließen.

### Einfluss der Inzidenz von SARS-CoV-2 in der Allgemeinbevölkerung

Hohe Inzidenzen innerhalb der Bevölkerung führen zu einer Erhöhung von SARS-CoV-2-Übertragungen im Schulbetrieb [10]. Wie stark dieser Zusammenhang ist, zeigt eine Studie aus England. Das Risiko eines Ausbruchs im Schulbetrieb stieg in dem untersuchten Setting mit jedem Anstieg von 5/100.000 Fällen in der Bevölkerung um 72 % an [37]. Allerdings dürfte nach mehreren Analysen hier das Lehrpersonal eine größere Rolle spielen als die Schüler und Schülerinnen [7]. Tatsächlich zeigen Untersuchungen aus den USA, dass auch bei hoher Umgebungsinzidenz Übertragungen in den Schulen bei entsprechenden Hygienevorkehrungen sehr selten sind [38]. Die Hygienekonzepte sind dabei jedoch sehr wichtig: Studien, in denen die Rolle der Schulen betrachtet wurde, ohne dass diese adäquate Hygienekonzepte umgesetzt hatten, zeigen, dass dann Übertragung eher möglich ist [39]. Primär sollte also die Verbreitung des Virus in der Gesamtbevölkerung eingedämmt werden sowie entsprechende Hygienekonzepte zur konsequenten Anwendung kommen. So kann verhindert werden, dass das Virus in die Schule getragen wird [10].

### Keine relevante Beeinflussung des Infektionsgeschehens an Schulen durch neue Virusvarianten

Als in England erstmals von der ansteckenderen Virusvariante B.1.1.7 (Alphavariante) zu hören war, war die Sorge groß, dass diese neue Virusmutante auch das Infektionsgeschehen an Schulen negativ beeinflussen könnte. Dies führte wiederum zu flächendeckenden Schulschließungen in der zweiten und dritten Pandemiewelle. Die aktuelle Datenlage liefert hierfür jedoch keine Hinweise. Österreich führt mit der sogenannten Gurgelstudie eine Kohortenstudie in 5 % der österreichischen Schulen in der Altersklasse 6–14 Jahre durch [40]. In der dritten Erhebungsphase im März 2021, in der ein Großteil der Neuinfektionen bereits auf die Mutation B.1.1.7 zurückzuführen war, lag die Prävalenz von SARS-CoV‑2 mit 0,21 % unter der Prävalenz im November 2020 von 1,39 % und unter der Prävalenz im Oktober 2020 von 0,39 %.

Insgesamt hat sich auch die Befürchtung nicht bestätigt, dass die Alphavariante gehäuft bei Kindern und Jugendlichen auftritt und somit zu einem Problem an Schulen werden könnte [41].

### „Sozialer Deprivationsindex“ als Risikofaktor

Bei Analyse der Daten der Gurgelstudie hat man feststellen können, dass – wie auch bei Erwachsenen – eine Assoziation der SARS-CoV-2-Infektionsrate mit dem sozialen Deprivationsindex vorliegt [40]. Dies passt zu internationalen Daten, nach denen die Wahrscheinlichkeit einer SARS-CoV-2-Infektion in sozial benachteiligten Gruppen um etwa das 3‑Fache erhöht ist [42]. Wie auch im Oktober 2020 und November 2020 wurde in Schulen mit hoher/sehr hoher sozialer Benachteiligung eine höhere Prävalenz als in Schulen mit einem Index geringer/moderater sozialer Benachteiligung nachgewiesen. Dies deutet auf den Einfluss des die Schule umgebenden sozialen Milieus hin und somit auf vermehrte Eintragung von Infektionen in die Schulen bei entsprechender äußerer Umgebung.

### Auswirkungen flächendeckender Testung an Schulen

Seit den Osterferien 2021 besteht an den meisten Schulen in Deutschland eine zweimal wöchentliche Testpflicht, um am Präsenzunterricht in der Schule teilnehmen zu können, in manchen Regionen begann diese auch schon früher. Der Einfluss dieser Teststrategie wurde in einem Artikel des Gesundheitsamts Frankfurt am Main beleuchtet [36]. Es wurden die Inzidenzen in der Gruppe der 5‑ bis 9‑Jährigen und der 10- bis 14-Jährigen nach den Osterferien 2021 analysiert. In der ersten Woche nach den Osterferien, Kalenderwoche (KW) 16, stiegen die Inzidenzen in der oben genannten Altersgruppe rapide auf 233/100.000. Nachdem viele Landkreise wegen Inzidenzen von über 165/100.000 wieder in den Distanzunterricht gewechselt hatten, sanken die Inzidenzen in den genannten Altersgruppen.

Die Autoren der Studie schlussfolgern daraus jedoch nicht, dass Schulen nun Treiber der Pandemie waren. Vielmehr bringen sie den Anstieg der Inzidenz bei Kindern mit der geänderten Teststrategie in Verbindung. Letztendlich lag zum ersten Mal eine nahezu 100 % umfassende Stichprobe in der Altersgruppe vor, für die die Testpflicht in Kraft trat. Da für Erwachsene diese umfangreiche Stichprobe nicht vorlag, kann man nicht daraus schließen, dass Kinder häufiger mit SARS-CoV‑2 infiziert waren. Außerdem betonen die Autoren, dass bei einem Anstieg der positiven Testergebnisse in der Woche direkt nach den Osterferien die Infektionen höchstwahrscheinlich nicht in der Schule, sondern aufgrund von Inkubationszeiten in der vorhergegangenen Woche innerhalb der Ferien erworben wurden.

### Der Impffortschritt und seine Auswirkungen auf Schulen

Aktuell gibt es mehrere zugelassene Impfstoffe gegen SARS-CoV‑2 in Deutschland, allerdings sind die meisten erst ab dem 16. bzw. 18. Lebensjahr zugelassen. Seit Juni 2021 besteht eine Zulassung des Impfstoffs von BioNTech/Pfizer sogar schon ab dem 12. Lebensjahr. Allerdings wird die Impfung gegen SARS-CoV‑2 mit Stand von Juni 2021 von der Ständigen Impfkommission (STIKO) nur für Kinder mit relevanten Vorerkrankungen empfohlen [43]. Trotzdem ist zu erwarten, dass die Impfung von immer mehr Erwachsenen sich auch positiv auf die Infektionszahlen bei Kindern auswirkt, da geimpfte Lehrkräfte als Vektoren für SARS-CoV‑2 an Schulen wegfallen und geimpfte Eltern ihre Kinder zu Hause vor einer Infektion schützen [44].

Tatsächlich stimmen Daten aus Israel optimistisch, dass die Impfung auch unter Alltagsbedingungen einen sehr guten Schutz gegen eine Infektion mit SARS-CoV‑2 bietet [45]. Bis zum Ende des Beobachtungszeitraums der Studie waren 72,1 % der Einwohner Israels über 16 Jahre mit der zweiten Dosis des Impfstoffes von BioNTech/Pfizer geimpft. Die Wirksamkeit des Impfstoffes wurde abschließend mit 95,3 % beurteilt. Die größte Wirksamkeit in allen Bereichen konnte in der Alterskohorte von 16–44 Jahren festgestellt werden. Es zeigte sich, dass im Rahmen des Impffortschritts die Inzidenz von SARS-CoV‑2 stetig abnahm. Auch bei den nicht geimpften Kindern waren in Israel die Zahlen deutlich gesunken, wahrscheinlich durch einen Herdeneffekt und die insgesamt niedrige Gesamtinzidenz. Tatsächlich lag die Sterblichkeit an SARS-CoV‑2 in Israel inzwischen fast bei null, ohne dass mit den Impfungen von unter 16-Jährigen begonnen wurde.

Eine ähnliche Entwicklung ist auch in Deutschland zu erwarten. Hier haben allerdings Stand 24.06.2021 erst 52 % der Bevölkerung eine Erstimpfung erhalten, 34 % waren bereits mit 2 Dosen geimpft [33]. Ob sich die Wirksamkeit der Impfung mit dem Auftreten neuer Varianten von SARS-CoV‑2 ändert, bleibt abzuwarten.

## Kontroversen und Probleme bei der Bewertung der aktuellen Studienlage

Die momentane Debatte um die Rolle der Kinder und Jugendlichen und der Schulen im Rahmen der Pandemie ist auch zu einem Teil durch erhebliche Defizite in wissenschaftlicher Datenanalyse und Datenbewertung gekennzeichnet [8].

Viele der anfänglichen Studien wurden während oder kurz nach Schulschließungen durchgeführt. Diese Aussagen können so möglicherweise nur eingeschränkt auf den normalen Schulbetrieb übertragen werden.

Auch können Maßnahmen nicht gänzlich unabhängig voneinander beurteilt werden und beobachtete Effekte nicht einer einzigen Maßnahme zugeordnet werden. So kann nicht ausgeschlossen werden, dass ein geringeres Ansteckungsrisiko während Schulschließungen auch mitbedingt ist durch die geringere Gesamtmobilität in dieser Zeit. Dem widerspricht jedoch, dass Studien, die sich mit Infektionen im familiären Umfeld beschäftigen, zu dem Ergebnis kommen, dass das Ansteckungsrisiko von Kindern deutlich unter dem der Erwachsenen liegt [2].

Die Beurteilung, inwiefern die Schließung von Schulen tatsächlich zu einer Bremsung des Infektionsgeschehens geführt hat, wird so erschwert. Während einige Studien nur einen geringen oder keinen Effekt messen können, wird in anderen (vor allem Modellierungs‑)Studien von einer hohen Wirksamkeit von Schulschließungen ausgegangen [46, 47].

Ein weiteres Problem ist die Unklarheit darüber, wie viele Fälle einer SARS-CoV-2-Infektion bei Kindern tatsächlich asymptomatisch verlaufen. Hierzu gibt es unterschiedliche Angaben. Möglicherweise führen asymptomatische Verläufe bei Kindern dazu, dass Übertragungen an Schulen in der Datenlage unterrepräsentiert sind [10]. Es ist jedoch anzunehmen, dass dies auf SARS-CoV-2-Infektionen in der gesamten Bevölkerung zutrifft und nicht nur auf die Kinder.

## Möglichkeiten der Optimierung der Teststrategie an Schulen

Um validere Daten zum Infektionsgeschehen bei Schulkindern zu erhalten, sollte vorerst eine valide Teststrategie gewählt werden. Die aktuell angewendeten Antigenschnelltests müssen hier sehr kritisch betrachtet werden. Angesichts einer sehr schlechten Sensitivität in der Anwendung im Routinebetrieb und einer gänzlich ungeprüften Sensitivität bei symptomlosen Kindern ist eine breite Anwendung in Schulen wissenschaftlich nicht zu begründen und nicht zu rechtfertigen. Wie eine sinnvolle Teststrategie aussehen könnte, sowie die Vor- und Nachteile verschiedener Teststrategien wurden in einer Stellungnahme der DGPI zusammengefasst (Stand 28.02.2021; [48]).

Bei einer wie auch immer durchgeführten flächendeckenden Testung von Schulkindern sollten die Ergebnisse dieser Testungen zentral erfasst werden, um das Infektionsgeschehen bei Kindern und Jugendlichen sehr genau beobachten, wissenschaftlich auswerten und lokal Maßnahmen der Bekämpfung rascher einleiten zu können. Die Ergebnisse der derzeit durchgeführten Selbst- bzw. Schnelltests werden aktuell überhaupt nicht erfasst. Diese Daten nicht zu nutzen, um z. B. das Testverfahren zu evaluieren und damit Schulen zu sichereren Orten in der COVID-19-Pandemie zu machen, erscheint als Verschwendung von Ressourcen zulasten der Schulkinder.

## Fazit

Zusammenfassend kann festgehalten werden, dass bis dato durchgeführte Studien mehrheitlich zeigen, dass SARS-CoV-2-Infektionen von Kindern meist aus dem außerinstitutionellen Bereich in Schulen eingetragen und dort nur selten übertragen werden. Ein relevanter Beitrag zum Infektionsgeschehen ist somit unwahrscheinlich. Zudem ist durch den Impffortschritt mit einer wachsenden Zahl an Geimpften ein Rückgang der Infektionen bei Kindern und damit auch an Schulen und Kindertagesstätten zu erwarten. Aus diesen Gründen sind generelle Schließungen von Schulen und Kindertagesstätten auf Basis der aktuellen Datenlage und auch angesichts der derzeit bekannten Virusmutationen weder zu begründen noch zu rechtfertigen und sollten das letzte Mittel in der Bekämpfung der Pandemie sein.

## Footnotes

1 Indexfall: erster diagnostizierter Fall in einer Infektionskette.
